# Rapid Production of Human VEGF-A following Intradermal Injection of Modified VEGF-A mRNA Demonstrated by Cutaneous Microdialysis in the Rabbit and Pig* In Vivo*

**DOI:** 10.1155/2019/3915851

**Published:** 2019-01-15

**Authors:** Susanne Pehrsson, Mikko Hölttä, Gunilla Linhardt, Regina Fritsche Danielson, Leif Carlsson

**Affiliations:** ^1^Bioscience Heart Failure, Cardiovascular Renal and Metabolism, IMED Biotech Unit AstraZeneca Gothenburg, Sweden; ^2^IMED Biotech Unit, Drug Safety and Metabolism, Mechanistic Safety and ADME Sciences, AstraZeneca R&D, Gothenburg, Sweden; ^3^Cardiovascular, Renal and Metabolism, IMED Biotech Unit, AstraZeneca, Gothenburg, Sweden

## Abstract

Chemically modified mRNA is a novel, highly efficient, biocompatible modality for therapeutic protein expression that may overcome the challenges and safety concerns with current gene therapy strategies. We explored the efficiency of intradermally injected modified VEGF-A_165_ mRNA (VEGF-A mRNA) formulated in a biocompatible citrate/saline buffer to locally produce human VEGF-A_165_ protein. Rabbits (n=4) and minipigs (n=3) were implanted with subcutaneous microdialysis probes close to the injection sites and interstitial-fluid samples and skin biopsies were analysed for production of VEGF-A protein over time for up to 8 hours. Three to 4 hours after the intradermal injection of VEGF-A mRNA, detectable levels of human VEGF-A protein were seen in the microdialysis eluates in both species. In the pig, the VEGF-A concentrations increased dose-dependently reaching a maximum 6 hours after dosing (62.7±28.4, 357.6±240.6, and 746.3±210.2 pg/mL following injection of 24, 120, and 600 *μ*g VEGF-A mRNA, respectively). Likewise, in tissue biopsies harvested at study end (8 hours after VEGF-A mRNA injection), the content of VEGF-A protein increased dose-dependently. In contrast, VEGF-A protein was not detected in eluates originating from sites injected with citrate/saline vehicle. It is concluded that intradermal injection of VEGF-A mRNA is associated with a rapid and local production of VEGF-A protein. Considering the pro-angiogenic effect of VEGF-A, VEGF-A mRNA may hold promise for regenerative treatment of patients with diabetic wounds and ischemic cardiovascular disease.

## 1. Introduction

Through stimulation of angiogenesis, arteriogenesis, and lymphangiogenesis, therapeutic vascular growth is emerging as a concept for management of vascular indications such as coronary and limb ischemia and chronic wounds [1]. The vascular endothelial growth factor (VEGF) family members are key regulators of vascular growth and VEGF-A administered as recombinant protein or via gene transfer has received much attention for its potential beneficial effects in cardiovascular medicine. However, randomized controlled trials have not lived up to expectations and evidence of clinical efficacy is inconclusive. The reason(s) for the lack of clinical benefit is currently somewhat unclear but may include factors such as suboptimal pharmacokinetics and local concentrations of the protein, poor gene transfer efficiency, inappropriate dosing, and regression of immature vessels as well as growth factor-related adverse effects and prolonged expression of the paracrine factor. To circumvent the issues associated with recombinant protein and gene transfer modalities, synthetic and chemically modified mRNA has been put forward as a non-immunogenic and non-integrating modality for transient expression of proteins in mammalian cells [2–4]. However, prior* in vivo* work with modified VEGF-A mRNA (VEGF-A mRNA) therapies has relied on cationic lipid carriers and non-optimized mRNA transcript production methods that may cause infusion-related hypersensitivity reactions, can be associated with tissue injury, and promote RNA degradation via modulation of the local innate immune system [5]. Recent observations indicate, however, that, by direct injection in cardiac and skeletal muscle, local delivery of modified mRNA formulated simply in citrate-buffered saline may result in sustained and efficient transfection of the mRNA [6–8]. Hence, in mouse, rat, and pig models of myocardial infarction, intramyocardial injection of VEGF-A mRNA led to elevated cardiac VEGF-A protein levels and improved heart function and survival, which were associated with improved formation of new blood vessels around the infarct [6, 7]. Furthermore, when given intradermally in a mouse ear model, VEGF-A mRNA induced vasodilation, blood flow increase, capillary angiogenesis, and neovascularization [9]. Prompted by these encouraging experimental findings, VEGF-A mRNA is currently undergoing clinical testing in type 2 diabetic patients and in patients undergoing coronary artery by-pass grafting [10–12]. The present series of* in vivo *experiments is the first to demonstrate that intradermal injection of VEGF-A mRNA formulated in a biocompatible buffer results in local production of human VEGF-A protein as demonstrated via serial interstitial-fluid sampling and analysis of eluates from cutaneous inserted microdialysis probes.

## 2. Materials and Methods

### 2.1. Preparation of Rabbits for Drug Administration and Microdialysis

Four male New Zealand White rabbits (2.4-3.0 kg body weight, Lidköpings rabbit farm, Sweden) were anaesthetised with ketamine (5 mg/kg, Ketalar®, Pfizer AB, Sollentuna, Sweden) and medetomidine (0.15 mg/kg, Domitor®, Orion Pharma, Espoo, Finland) administered as an intravenous bolus injection followed by a maintenance infusion (11 and 0.33 mg/kg*∗*h), respectively. The rabbits were intubated and artificially ventilated with a mixture of room air and 10% O_2_ with a Servo ventilator (900D, Siemens Elema, Solna, Sweden). The respiratory rate was kept constant at 30 cycles/min. Before and during the experiment, the blood gases and pH in arterial blood were measured by a blood gas analyzer (ABL800 Flex, Radiometer, Copenhagen, Denmark) and, if necessary, adjusted to fall within normal physiological ranges for rabbits by adjusting the tidal volume. The rectal temperature was kept between 38 and 39.5°C by covering the animals and by external heating.

A percutaneous polyethylene catheter (Venflon 0.8 mm, Viggo, Helsingborg, Sweden) for administration of anaesthetics was inserted into a marginal vein on the left ear. A polyethylene catheter (Intramedic PE-90 Clay Adams, Becton Dickinson, Sparks. MD, USA) was inserted into the right carotid artery for arterial blood pressure recording (by means of a pressure transducer, Peter von Berg Medizintechnik Gmbh, Kirchseeon/Englharting. Germany) and for blood sampling, respectively. Signals from blood pressure measurements were recorded and sampled by using a computer and software (PharmLab V6.6, AstraZeneca R&D Mölndal, Sweden). The fur on the left hind leg was removed with an electric razor.

Two 100 kDa linear microdialysis probes, named A and B in each experiment (66 linear catheter & 66 high cut off linear catheter, M Dialysis AB, Hammarby, Stockholm, Sweden), were inserted id on the upper part of the left hind leg of the rabbit according to the instructions provided by the supplier. The microdialysis probes were perfused with 0.5 *μ*L/min physiological saline (9 mg/mL, Fresenius Kabi AG, Bad Homburg, Germany) and the eluate samples were collected on ice in pre-weighed 0.5 mL tubes (Protein LoBind, Eppendorf AG, Hamburg, Germany). Dead space between the dialysis membrane and the collection tube outlet was about 1.5 *μ*L. The volume of each eluate was determined and 2% bovine serum albumin (BSA, A7979, Sigma-Aldrich, St. Louis, MO, USA) in phosphate buffered saline (PBS pH 7.4, gibco® by life technologies™, Paisley, UK) was added at 1:1 conditions. The samples were stored at -80°C until analyzed.

**Figure 1:**
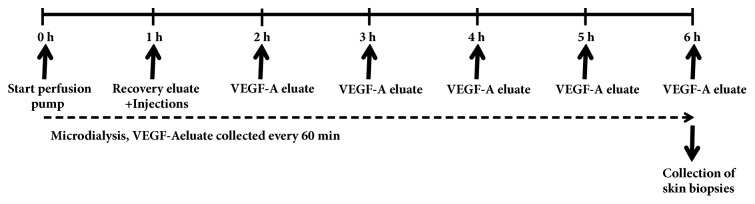
Experimental flow chart in the rabbit study. For further details, see methods.

**Figure 2:**
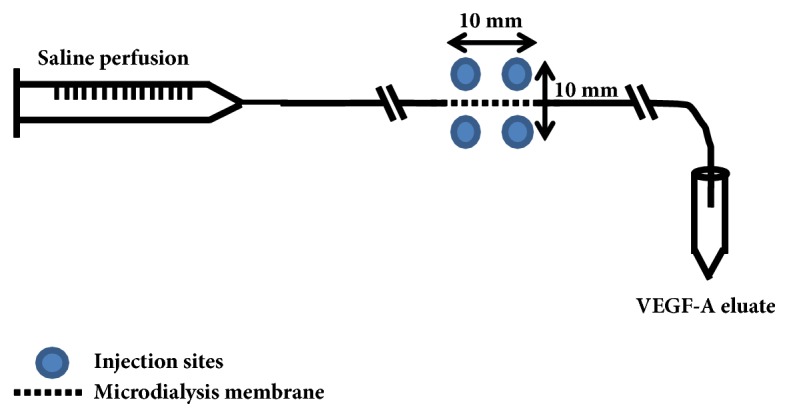
Placement of VEGF-A mRNA injections in relation to the microdialysis probe in the rabbit study. For further details, see methods.

The experimental design is illustrated in Figure 1. At t=0 h, the recovery period for both microdialysis probes was started. One hour later (i.e., t=1 h), recovery eluate was collected and subsequently 4 intradermal injections of VEGF-A mRNA formulated in citrate/saline buffer (10 mmol/L/130 mmol/L, pH 6.5) at the microdialysis-probe sites were given as depicted in Figure 2. Protein-containing eluate was collected every hour from t=2 h to t=6 h and handled as described above. At study end, the animals were terminated by a lethal iv dose of pentobarbital sodium (Allfatal vet, Omnidea AB, Stockholm, Sweden).

### 2.2. Preparation of Pigs for Drug Administration and Microdialysis

Three female Göttingen minipigs (22-26 kg body weight, Ellegaard, Dalmose, Denmark) were premedicated with intramuscular ketamine (10 mg/kg, Ketalar®, Pfizer AB, Sollentuna, Sweden) and midazolam (2 mg/kg, Dormicum®, Roche AB, Stockholm, Sweden). After approximately 20 min, a polyethylene catheter (Venflon 1.0 x 32 mm, Becton Dickinson, Helsingborg, Sweden) was inserted into an ear vein for administration of anaesthetics, a bolus dose of propofol (approximately 2 mg/kg, Propofol®, Lipuro, B. Braun Melsungen AG, Melsungen, Germany). The pigs were intubated and the anaesthesia was maintained with a subsequent intravenous infusion of propofol (2 mg/kg**∗**h during surgery and approximately 0.6 mg/kg*∗*h thereafter) complemented by 2 to 3% isoflurane (Isoba® vet, Schering-Plough, Denmark). The pigs were ventilated (Servo Ventilator 900C, Siemens Elema, Solna, Sweden) with room air supplied with 10% O_2_. Tidal volume and respiration rate were adjusted to maintain arterial blood gases and pH within normal physiological ranges for the species, which were checked repeatedly during the experiment by a blood gas analyzer (ABL800 Flex, Radiometer, Copenhagen, Denmark). Rehydrex (5 mL/kg*∗*h, Fresenius Kabi AS, Halden, Norway) was given iv continuously to replace fluid loss. The rectal temperature was kept between 35.6 and 37.1°C by covering the animals and by external heating.

Two Seldinger introducers (French 6, Radifocus® Introducer II, Terumo Corporation, Tokyo, Japan) were inserted in the right femoral artery for collection of blood and for continuous registration of mean arterial blood pressure and heart rate and in the right femoral vein in case an extra vein access was needed. An electrocardiogram was recorded from skin electrodes. Signals were recorded by using a customized computer and software system (PharmLab V6.0, AstraZeneca R&D Gothenburg, Sweden).

**Figure 3:**
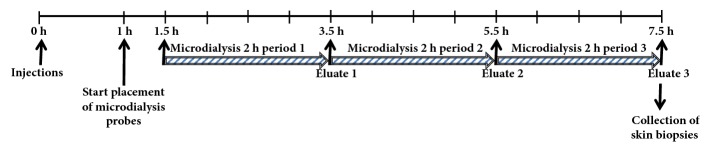
Experimental flow chart in the pig study. For further details, see methods.

**Figure 4:**
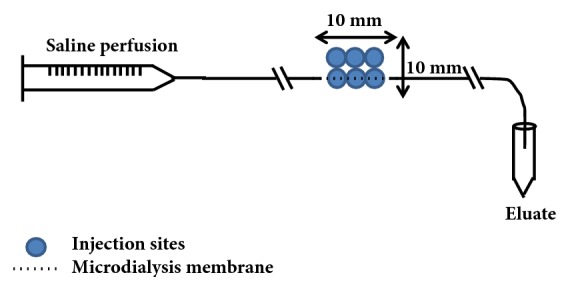
Placement of VEGF-A mRNA injections in relation to the microdialysis probe in the pig study. For further details, see methods.

The experimental design is illustrated in Figure 3. At t=0 h, VEGF-A mRNA or citrate/saline vehicle was intradermally injected at the intended microdialysis-probe sites as depicted in Figure 4. One h later (i.e., t=1 h), the microdialysis-probes were implanted and perfusion was initiated. Protein-containing eluate was collected during 2-hour periods starting at t=1.5 hours for up to t=7.5 hours after the study drug was injected and handled as described above. At approximately 8 hours after the intradermal injections, the injection sites were harvested and the skin biopsies were stored at -80°C until analyzed. At the end of the experiment, the animals were terminated by a lethal intravenous dose of pentobarbital sodium (Allfatal vet, Omnidea AB, Stockholm, Sweden).The materials and methods section should contain sufficient detail so that all procedures can be repeated. It may be divided into headed subsections if several methods are described.

Six intradermal injections at each intended microdialysis-probe site on the abdomen of the pig were given in two rows with three injections on each side as illustrated in Figure 4. Six 100 kDa linear microdialysis probes, (66 linear catheter & 66 high cut off linear catheter, M Dialysis AB, Hammarby, Stockholm, Sweden) were inserted intradermally according to the instructions provided by the supplier, along one of the two injection rows (Figure 4). The microdialysis probes were perfused with 0.5 *μ*L/min physiological saline (9 mg/mL, Fresenius Kabi AG, Bad Homburg, Germany) and the eluate samples were collected on ice in pre-weighed 0.5 mL tubes (Protein LoBind, Eppendorf AG, Hamburg, Germany). Dead space between the dialysis membrane and the collection tube outlet was about 1.5 *μ*L. The volume of each eluate sample was measured and bovine serum albumin (BSA, A7979, Sigma-Aldrich, St. Louis, MO, USA) in phosphate-buffered saline (pH 7.4, Gibco® by Life Technologies™, Paisley, UK) was added at 1:1 conditions to a final concentration of 2% albumin. The samples were stored at -80°C until analyzed.

### 2.3. Assessment of Human VEGF-A Protein in Microdialysis Eluates

The Gyrolab platform was used for determination of levels of expressed human VEGF-A protein in the microdialysis eluate samples from the rabbit. The Gyrolab uses an affinity flow-through format with microstructure wells (Gyros, Uppsala, Sweden). A Gyrolab bioaffy 1000 CD consisting of 96 microstructure wells containing an affinity capture column with streptavidin coated material (Gyros) was used. First, a biotinylated capture polyclonal antibody against human VEGF A (AF-293-NA, R&D systems, Abingdon, UK) was immobilized on the streptavidin column, where samples are flowed through by rotation gravity and the analyte is captured on the antibodies. An Alexa-labelled detection antibody against human VEGF-A (R&D systems) was then flowed through the column and the florescence intensity was used for quantification of the ligand. A standard curve was created using a five-parametric linear fit and the sample concentrations were calculated from the standard curve according to their absorbance. A standard curve ranging from 16.7 pg/mL to 12170 pg/mL was prepared with human VEGF-A_165_ (293-VE-010, R&D systems) in MSD diluent 9 (Meso Scale Discovery, Rockville, Maryland, USA). Quality controls were prepared from the WHO standard of human VEGF-A_165_ (National Institute for Biological Standards and Control, Hertfordshire, UK) in MSD diluent 9. All standard, QCs, and samples were mixed 1:1 with Rexxip HN-max (Gyros) before analysis. Lower Limit of Quantification (LLOQ) was 33.4 pg/mL.

Concentrations of VEGF-A165 in the pig microdialysates were determined using a sandwich immunoassay with electrochemical luminescent detection. V-PLEX Human VEGF assay kit (K151RHD, Meso Scale Diagnostics) was used to measure the VEGF-A165 concentration in the microdialysates. Standards were serially diluted in MSD diluents. Samples were diluted 4x with MSD diluents prior to analysis, and the plates were read on the MSD's Sector Imager 6000. LLOQ was 15.6 pg/mL

### 2.4. Assessment of Human VEGF-A Protein In Pig Skin Biopsies

Pig skin pieces from the injection sites were thawed and placed in Precellys tubes. A hydrochloric acid solution containing bovine serum albumin was added to the tubes and the samples were incubated overnight at 55°C. The samples were then neutralised with sodium hydroxide, where after MSD Tris lysis buffer with 2xTris was added. Stainless steel beads (2.8 mm) were placed in the sample tubes, and the samples were homogenized using the Precellys homogenizer. The homogenates were centrifuged, and the supernatants were stored at -80°C pending analysis. Concentrations of VEGF-A165 were determined using a sandwich immunoassay with electrochemical luminescent detection. V-PLEX Human VEGF assay kit (K151RHD, Meso Scale Diagnostics) was used to measure the VEGF-A165 concentration in the tissue homogenates. Standards were serially diluted in MSD diluents. Samples with high concentration were diluted with MSD diluents prior to analysis to fit within the standard curve, and the plates were read on the MSD's Sector Imager 6000. LLOQ was 0.28 pg/mg.

### 2.5. VEGF-A mRNA Drug Substance and Formulation

VEGF-A mRNA was manufactured by Moderna Therapeutics Inc. as previously described [6, 13]. Briefly, mRNA was synthesized in vitro using T7 polymerase-mediated transcription from a linearized DNA template containing the VEGF-A open reading frame, flanking 5′ and 3′ untranslated regions and a poly-A tail. A Cap1 structure was enzymatically added to the 5′ end to produce the final mRNA. Uridine was completely substituted with N1-methylpseudouridine to reduce potential immunostimulatory activity and to improve VEGF-A protein expression [6] relative to unmodified mRNA. After purification (purity 98.3%), the mRNA was diluted in the desired buffer and frozen at -20°C. A stock solution of VEGF-A mRNA was prepared at 2 mg/mL dilution of the thawed drug substance solution with a buffer solution containing 10 mmol/L sodium citrate dihydrate and 130 mmol/L sodium chloride, pH 6.5. The solution was sterilized by membrane filtration and filled into sterile vials. Further dilutions were done with the same buffer solution.

## 3. Results

**Figure 5:**
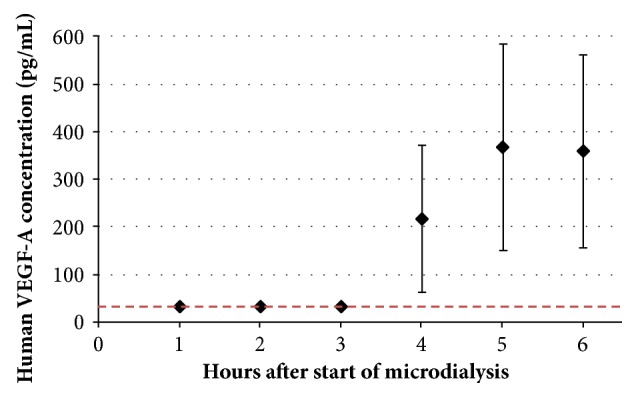
Concentrations of human VEGF-A in eluates from 100 kDa microdialysis probes intradermally inserted in the rabbit hind leg. Microdialysis was started at t=0 h and four id injections of VEGF-A mRNA injections (50 *μ*g each) were given at t=1 h. Dotted line indicates Lower Limit of Quantification (LLOQ, 33.4 pg/mL). Two probes were inserted in each rabbit. Values shown are mean ± SEM (n=4).

The human VEGF-A protein levels in eluates from the four rabbits (with two inserted probes each, A and B) are presented in Figure 5. Three hours after the injections of VEGF-A mRNA, detectable levels (218±155 pg/mL) of human VEGF-A protein were found in eluates from 3 out of the 8 probes. Correspondingly, at 4 and 5 hours after the injection, VEGF-A protein was detected in the eluate from 5 out of 8 and 5 out of 8 probes, respectively. Despite large variation in the concentrations observed, the protein levels tended to plateau at these time points (369±217 and 360±203 pg/mL, respectively).

**Figure 6:**
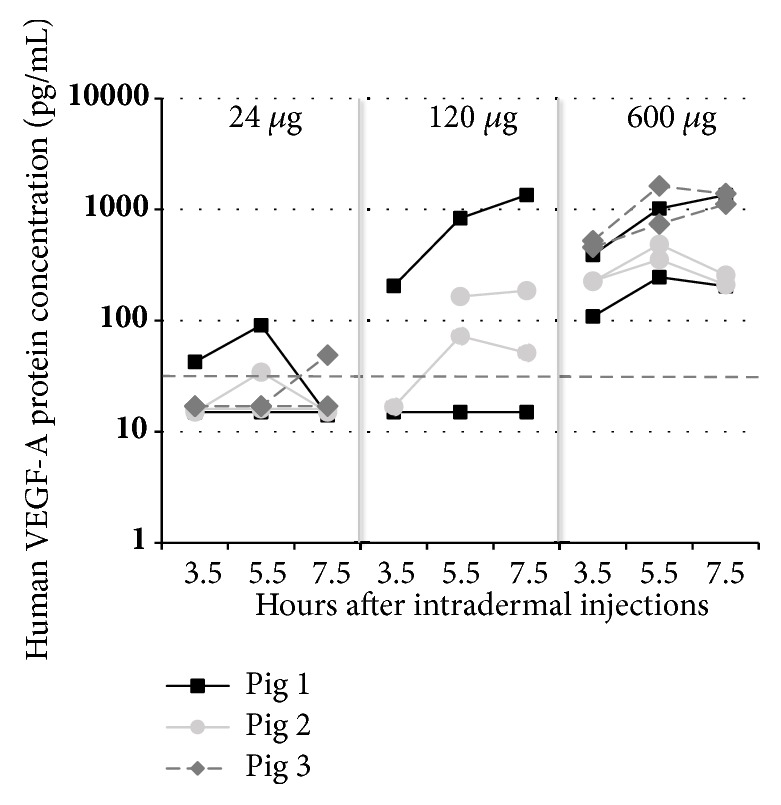
Concentrations of human VEGF-A in microdialysis eluates from 100 kDa microdialysis probes intradermally inserted on the pig (n=3) abdomen. Values presented as individual values in eluate from each probe. Six intradermal VEGF-A mRNA injections (50 *μ*L each, total 24, 120, or 600 *μ*g VEGF-A mRNA) were given at t=0 h. At t=1.5 h after the injections, microdialysis was started. Eluates were collected during the time intervals from 1.5 h to 3.5 h, from 3.5 h to 5.5 h, and from 5.5 h to 7.5 h, respectively. Dotted vertical line indicates Lower Limit of Quantification (33.4 pg/mL); eluate samples below LLOQ are depicted as 0.5*∗*LLOQ=16.7 pg/mL. Six probes were inserted in each pig, two probes per dose except the 120 *μ*g dose, where only four probes were used to assess human VEGF-A production following injection of 24 or 600 *μ*g VEGF-A mRNA. The remaining two probes were used to study effects following injection of citrate/saline vehicle.

**Figure 7:**
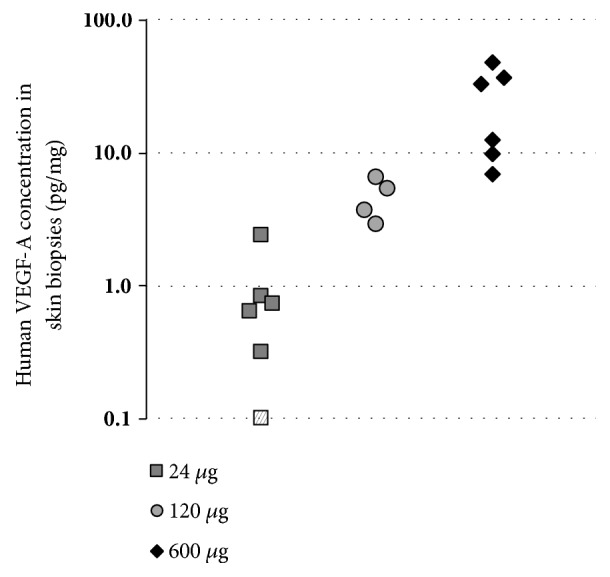
Amounts (pg/mg) of human VEGF-A in skin biopsies excised approximately 8 hours after intradermal injection of VEGF-A mRNA in 3 pigs. Each dose was administered as 6 (24 and 600 *μ*g dose) or 4 (120 *μ*g dose) separate injections at two different sites in each pig. Hatched data point in the 24 *μ*g dose, depicted at 0.1 pg/mg, indicating amount below Lower Limit of Quantification (0.28 pg/mg).

Human VEGF-A protein levels in eluates from each of the three individual pigs (with six inserted probes each, labelled A to F) are visualized in Figure 6. Three and a half hours after the injections of VEGF-A mRNA, detectable levels (42.5, 205.4 and 321.8±65.2 pg/mL, mean±SEM) of human VEGF-A protein were found in eluates from 1 of 6, 1 of 3 and 6 of 6 probes from the 24, 120 and 600 *μ*g dosing, respectively. Five and a half hours and seven and a half hours after the injections, the corresponding results were 62.7±28.4, 357.6±240.6, and 746.3±210.2 pg/mL in eluates from 2 of 6, 3 of 4, and 6 of 6 probes, and 48.8, 529.1±412.1, and 753.0±240.0 pg/mL in eluates from 1 of 6, 3 of 4, and 6 of 6 probes, respectively. Human VEGF-A protein was not detected in eluates from probes implanted at sites injected with citrate/saline vehicle at any time point. In accordance with the VEGF-A concentrations in the microdialysis eluates, the concentration of VEGF-A in the skin biopsies increased dose-dependently (1.0±0.4, 4.6±0.8, and 24.3±6.9 pg/mg for the 24, 120, and 600 *μ*g doses, respectively, Figure 7).

## 4. Discussion

The present series of experiments demonstrate that intradermal injection of synthetic chemically modified VEGF-A mRNA results in rapid local production of VEGF-A protein as shown by the concentration-dependent increase in VEGF-A protein in eluates from the interstitial microdialysis sampling. We used a human purified mRNA transcript with optimized capping efficiency, UTR, nucleotide design that currently is explored in clinical studies for its safety tolerability and efficacy [6, 10–12]. Recent observations in rats, pigs, and non-human primates indicate that this optimized construct when intracardially or intradermally injected results in rapid, efficient, and sustained transfection of VEGF-A protein [6, 9]. VEGF mRNA uptake and protein expression were followed by* in situ* hybridization and immunohistochemistry following intracardiac and intradermal injection. Within hours, uptake of mRNA and protein production where demonstrated to occur predominantly in cardiomyocytes, endothelial cells, and adipocytes and VEGF-A protein was produced for several days [6, 9]. Furthermore, the administration was not associated with activation of the innate immune system as evidenced by lack of increase in IL-6 or IL-8 when assessed 3 to 24 hours after injection. In accordance with the recent observations of efficient transfection and protein production of VEGF-A following intracardiac delivery of VEGF-A mRNA using a biologically compatible buffer thus avoiding use of lipid carriers, we found this citrate-saline formulation effective also when the mRNA was administered in the intradermal space [6]. An additional advantage of the biocompatible formulation is that the naked mRNA will be tissue specific producing the paracrine protein locally, thus avoiding systemic exposure as any mRNA escaping systemically will promptly be degraded by RNAse. Hence, citrate/saline-formulated VEGF-A mRNA administered intradermally or intravenously did not gave rise to any systemic levels of VEGF-A in rats or cynomolgus monkeys [6]. Taken together these important improvements have been instrumental in advancing this therapeutic modality into clinical testing to unlock its potential in ischemia-related disease.

In the present study we took the advantage of applying cutaneous microdialysis, which is a semi-invasive method to assess the magnitude and time course of exogenous or endogenous substances in the skin* in vivo*, to demonstrate production of human VEGF-A from intradermally administered VEGF-A mRNA. Detectable levels of VEGF-A protein were seen in the microdialysis eluates at 3 to 4 hours after the administration and tended to plateau at 6 to 8 hours. This time profile of uptake, translation, and release of modified mRNA-encoded proteins is similar to what has been reported in cardiac tissue homogenates [6, 8]. For practical and ethical reasons, the present study did not allow us to follow the VEGF-A protein production for extended periods of time but, in the heart, a single injection of VEGF-A mRNA was associated for protein production for up to 8 days in the rat [6]. In the present pig experiments, we were able to study the dose-dependency of the injected VEGF-A mRNA in each separate pig. Hence, 3 different doses (24, 120 and 600 *μ*g) were administered and were demonstrated to result in a concentration-dependent increase in protein in the eluates as well as in the skin biopsies harvested at study termination. This is partly in contrast to the apparent lack of dose-dependency seen in cardiac tissue. Hence, Sultana and colleagues did not find any significant differences in the amounts of protein produced in transgenic mice intracardially injected with modified Cre mRNA and, in the study by Carlsson et al., increasing the dose of VEGF-A mRNA 10-fold increased the area under the curve by 1.6-fold only [6, 8]. The reason behind such a saturable uptake process in cardiac tissue and an almost linear protein production in the skin over a wide range of doses is currently unknown but may include saturable effects on the translation and secretion machineries as well.

The doses of VEGF-A mRNA injected in the present study are similar to doses previously administered to mice, rats, and pig and have resulted in functional effects which are in line with the well-known biological effects of VEGF-A on cardiovascular function in general and angiogenesis in particular. Hence, in mice, rats, and pig, single intracardiac injection of VEGF-A mRNA in the setting of acute or sub-acute myocardial infarction has been demonstrated to reduce infarct size, improve left ventricular function, and promote neovascularization [6, 7]. Furthermore, using the multi-parametric photoacoustic microscopy technique, single injections of VEGF-A mRNA in the mouse ear were demonstrated to cause marked and sustained upregulation of the local blood flow near the injection site. In addition, significant capillary angiogenesis and neovascularization 7 to 14 days after the injection of VEGF-A mRNA were noted [9]. However, although injection of recombinant VEGF-A protein was associated with acute cardiovascular effects, the changes were transient, and neovascularization was not observed in these animals.

In summary, we have demonstrated that a single intradermal injection of a modified VEGF-A mRNA in a biocompatible formulation results in a rapid and local production of VEGF-A indicating that this new modality may be of therapeutic benefit in patients with diabetic wounds and ischemic cardiovascular disease.

## Data Availability

The data that support the findings of this study are available from AstraZeneca, but restrictions apply to the availability of these data, which were used under license for the current study, and so are not publicly available. Data are available however from the authors upon reasonable request and with permission of AstraZeneca.
